# Designing an overview Theory of Change for a multi-component support community for people affected by rare dementia

**DOI:** 10.3389/frdem.2025.1565277

**Published:** 2025-06-06

**Authors:** Mary Pat Sullivan, Paul M. Camic, Emma Harding, Joshua Stott, Gill Windle, Ian Davies-Abbott, Sebastian J. Crutch

**Affiliations:** ^1^Faculty of Education and Professional Studies, School of Social Work, Nipissing University, North Bay, ON, Canada; ^2^Institute of Neurology, Dementia Research Centre, University College London, London, United Kingdom; ^3^Department of Clinical, Educational and Health Psychology, University College London, London, United Kingdom; ^4^Ageing and Dementia @ Bangor, School of Health Sciences, Dementia Services Development Centre, Bangor University, Bangor, United Kingdom; ^5^Faculty of Health Studies, The Centre for Applied Dementia Studies, University of Bradford, Bradford, United Kingdom

**Keywords:** rare dementia, young onset dementia, Theory of Change, program theory, rare dementia support, support community, biosociality, biosolidarity

## Abstract

**Introduction:**

There is growing awareness of people living with diverse dementia syndromes, many of whom are younger in age, with distinct support needs. Planning for increasing numbers of people living with dementia and subsequent models of support has largely overlooked this population. To address this gap, the aim was to design a Theory of Change for multi-component rare dementia support.

**Methods:**

Intervention development frameworks underpinned the construction of a Theory of Change informed by research evidence on rare dementia support and an iterative consultation process with people with lived experience, researchers, educators and health and social care practitioners.

**Results:**

The Theory of Change illustrates pathways to activities for continuous and tailored support solutions, education and knowledge production. Characteristic features include relationship, connection and continuity for people with lived experience, training and networking for professionals, and relational support with a commitment to ongoing learning for the rare dementia support team.

**Conclusion:**

The Theory of Change is positioned to flexibly support people affected by rare dementia, strengthen capacity within all sectors, improve service quality whilst maintaining a commitment to knowledge production and mobilization.

## 1 Introduction

Recent global prevalence rates indicate that by 2030 there will be an estimated 78 million people living with dementia (World Health Organization, 2021). The disease burden and consequent impact on care systems, including families, is well documented. National dementia strategies alongside the amplified voice of lived experience have stimulated a commitment to invest in and innovate service models or pathways to diagnosis and continuous multi-sectoral support. For example, the World Alzheimer Report (Alzheimer’s Disease International, 2022) which was dedicated to post-diagnostic support, highlighted the need to understand dementia symptoms and stages among diverse diseases or conditions, showcase pharmacological and non-pharmacological interventions and models of care, expand education for health professionals, and move forward person-centred and culturally appropriate support systems.

Over the last 25–30 years a vast number of studies have focused on dementia care, support and rehabilitation interventions that point to various opportunities to reduce burden and improve the quality of life for all those affected. Characteristic of much of this literature is a focus on memory-led dementia among an older population and the construction of generalist representations of people who are affected and dementia-related care and support services (Sullivan et al., 2023). The growth of support models that cater to an older population living with Alzheimer’s disease (AD) appropriately corresponds to our understandings of the impact of global ageing, age-related dementia risk, and much higher disease prevalence rates for AD in older age groups. However, with increased understandings of the neuropathology underlying dementia, there has been growing acknowledgement of both diverse dementia aetiologies and symptom presentation and corresponding diverse support needs. It is estimated, for example, that up to 15% of all dementia diagnoses are either young onset (i.e., symptom onset prior to the age of 65), have an autosomal dominant pattern of inheritance, or are non-memory-led forms of dementia with distinct symptom profiles (e.g., primary progressive aphasia, posterior cortical atrophy) (Graff-Radford et al., 2021; Hendriks et al., 2021; National Institute for Health and Clinical Excellence, 2024). There has also been more recent recognition that national dementia strategies and service development have largely overlooked populations such as those who live in rural areas, people from different ethno-racial communities and younger people diagnosed with dementia (Brijnath et al., 2022; Stamou et al., 2023; Wiese et al., 2023).

For individuals affected by a young onset dementia or those diagnosed with an atypical dementia characterized by distinct symptom profiles and more often young in onset, the transition to post-diagnostic support remains more complicated owing to still inadequate or inappropriate models of intervention (Rossi-Harries et al., 2024; Stamou et al., 2020). Therefore, for those who are affected by a rare dementia diagnosis there are currently fewer options for support that are catered to, for example, the underlying pathology and associated symptoms, illness stages, family status, and life stage and the associated psychosocial issues. In light of the numerous intersecting needs of these diverse populations it is critical to consider multicomponent interventions that address several needs simultaneously (e.g., psychoeducation, social support, care navigation, distress reduction). Such interventions are complex and need theoretical underpinning to inform further development, service planning, monitoring and evaluation.

As part of a 5-year multi-site study exploring multi-component support for people affected by rare dementia [Rare Dementia Support (RDS) Impact Study], we established a working group to explicate program or implementation theory for an innovative, theory-based and sustainable model of support. More specifically, our purpose was to translate the objectives of the emerging rare dementia support model (i.e., the program or framework for the program) to the support delivery (i.e., the implementation), for the purposes of planning and ongoing monitoring. Adopting a development approach in recognition of a constantly changing external environment (e.g., shrinking universal health care systems), we also valued the potential of program theory to inform longer term evaluation questions, building partnerships with other dementia organizations, and the localization of the rare dementia support program theory to other jurisdictions (Blamey and MacKenzie, 2007; Mayne, 2015). The aim of this paper is to describe the theoretical support model demonstrating a critical role for multi-component support for people affected by rare dementia, illustrative in the emerging Theory of Change (ToC), and the processes involved in its development.

## 2 Methods

### 2.1 Design

Admittedly, the literature on intervention evaluation is vast. Yet guidance on how researchers and practitioners can use theory, evidence and experience to practically develop a new program or intervention is much less accessible. Our work was broadly informed by the United Kingdom (UK) Medical Research Council’s updated Framework for Developing and Evaluating Complex Interventions (Skivington et al., 2021). We were guided by the Six Essential Steps for Quality Intervention Development (6SQuID) given its pragmatic focus and its flexible yet logical approach to achieving the development of a ToC. Designed for the development of public health interventions, 6SQuID emphasizes interdisciplinary research and practitioner collaboration, and co-production with service users and policymakers. The six steps to intervention development include: (i) defining and scoping the “problem”; (ii) clarifying causal and contextual factors that are malleable and have the greatest possibility for change; (iii) identifying the change mechanism; (iv) identifying how to deliver the change mechanism; (v) small scale testing and refining; and (vi) collecting evidence of effectiveness (Wight et al., 2016).

We also used Breuer and colleagues (Breuer et al., 2015) Checklist for Reporting Theory of Change in Public Health Interventions as a process guide as we progressed through 6SQuID. For the purposes of our work, ToC was defined as a process to think about support for people affected by rare dementia and a tool to describe a model of support and its impact, and how we expected to achieve the desired or intended change (Olsson et al., 2023).

### 2.2 Theoretical framework

Our work was underpinned theoretically by various sources within the literature illuminating the impact of dementia on individuals and families (e.g., dementia grief, care burden and negotiation, family functioning), access to specialist care, support and education, and peer engagement and support groups. In terms of mapping the support model the latter sources were instrumental in framing some of our overarching considerations. In addition, we drew on concepts and explanations that resonated within the research and support delivery we were undertaking at the time.

Through the integration of the disease and the social being, derives the concept of biosociality (Gibbon and Novas, 2008). Though not providing a thorough interrogation of its strengths and limitations here, biosociality reinforces the value of social spaces (communities) for people with shared biomedical conditions. Here, this new space or community provides an opportunity for those with a rare or stigmatized illness to share commonalities, learn from one another, share practical advice and potentially regain, as much as possible, a more normalized everyday life. Thought to shape both individual and collective identity, these communities provide an opportunity for members to exercise citizenship through relationships. This relatedness is also thought to inspire awareness raising and advocacy, or biosolidarity, and increase opportunities for others to connect and thereby strengthen the community and its activities (Bradley, 2021; Meleo-Erwin, 2020).

The concept of relational citizenship and relationship-centred care with support for people living with dementia and their active participation in their own care emerges in the more recent generation of dementia theorizing (Kontos et al., 2017). Similarly, relational safety and its complementary concepts of interpersonal competence and relational autonomy emerge in several studies addressing peer support for people living with dementia in support of the development of opportunities for social participation and inclusive spaces for people living with dementia (Sullivan et al., 2022).

Complementing these understandings is ecological systems theory and its strengths in relation to understanding the intersection of an individual’s microsystem (e.g., beliefs, values, patterns of activities, roles, resources) and the various other micro-systems around them, or the mesosystem (e.g., primary health care provider, neighbour, employer, support group facilitator) (Bronfenbrenner, 1986). We recognize the likelihood of increasingly complex intersections among microsystems for those affected by a rare dementia diagnosis, and the possibility for marginalization or isolation as the relevant systems reposition themselves in response to the diagnosis and everchanging care and support needs. These system interdependencies draw attention to the strengthening of each, through information and education or continuous support, for example, in recognition that systems will continue to navigate transitions and related adaptations as an individual’s dementia progresses (Crawford, 2020).

### 2.3 Setting

The research study was led by University College London Dementia Research Centre (UCL DRC) in the United Kingdom (UK). Having run a number of disease or syndrome specific support groups since the mid-1990s, the Centre established Rare Dementia Support (RDS) in 2016 to formalize its identity as a specialist dementia support program and draw on existing strengths and relationships to begin to expand support offerings to people affected by seven diseases or conditions: young onset AD, familial AD, frontotemporal dementia, familiar frontotemporal dementia, primary progressive aphasia, posterior cortical atrophy, and Lewy body dementia. Funding for RDS was provided by the National Brain Appeal – a charity that fundraises to advance treatment and research for people affected by neurological conditions. The term “rare” was adopted to highlight the symptoms that are often overlooked or unrecognized in common forms of dementia (e.g., variants of AD), and support strategies that were designed for or tailored to specific diseases and syndromes but also useful for anyone living with dementia. The multi-component RDS Impact Study (2019–2023) was timely in its facilitation of knowledge development specific to multi-component tailored support and the knowledge mobilization via the supports delivered by RDS (Brotherhood et al., 2020).

Adopting an iterative and participatory approach, we formed a working group (N = 7) from among the larger research team (N = 28) to begin the collaborative process of bringing together perspectives and knowledge to initiate the ToC development. Often referred to as “content experts” with an “inside lens” (Ghate, 2018), members of the working group included people affected by rare dementia, experienced clinical specialists, and interdisciplinary dementia researchers and educators (e.g., psychology, social work). Although the work primarily focused on the English context, the group included members from Wales and Canada given the fuller study included these countries as embedded case studies.

Bi-monthly working group meetings were held virtually on an encrypted video-conferencing platform for 20 months (2021–22). Meetings were audio-visually recorded and transcribed. Familiar in qualitative research methods, the transcripts were used for the purposes of establishing trustworthiness or validity of the “data” (i.e., thinking and conceptualizing) informing the emerging ToC (Morse, 2015). Principally, deliberations led to the development of a visual illustration of the ToC design that was used to structure and guide meeting discussions and refinement of the theory itself.

### 2.4 Data sources

**Figure 1:**
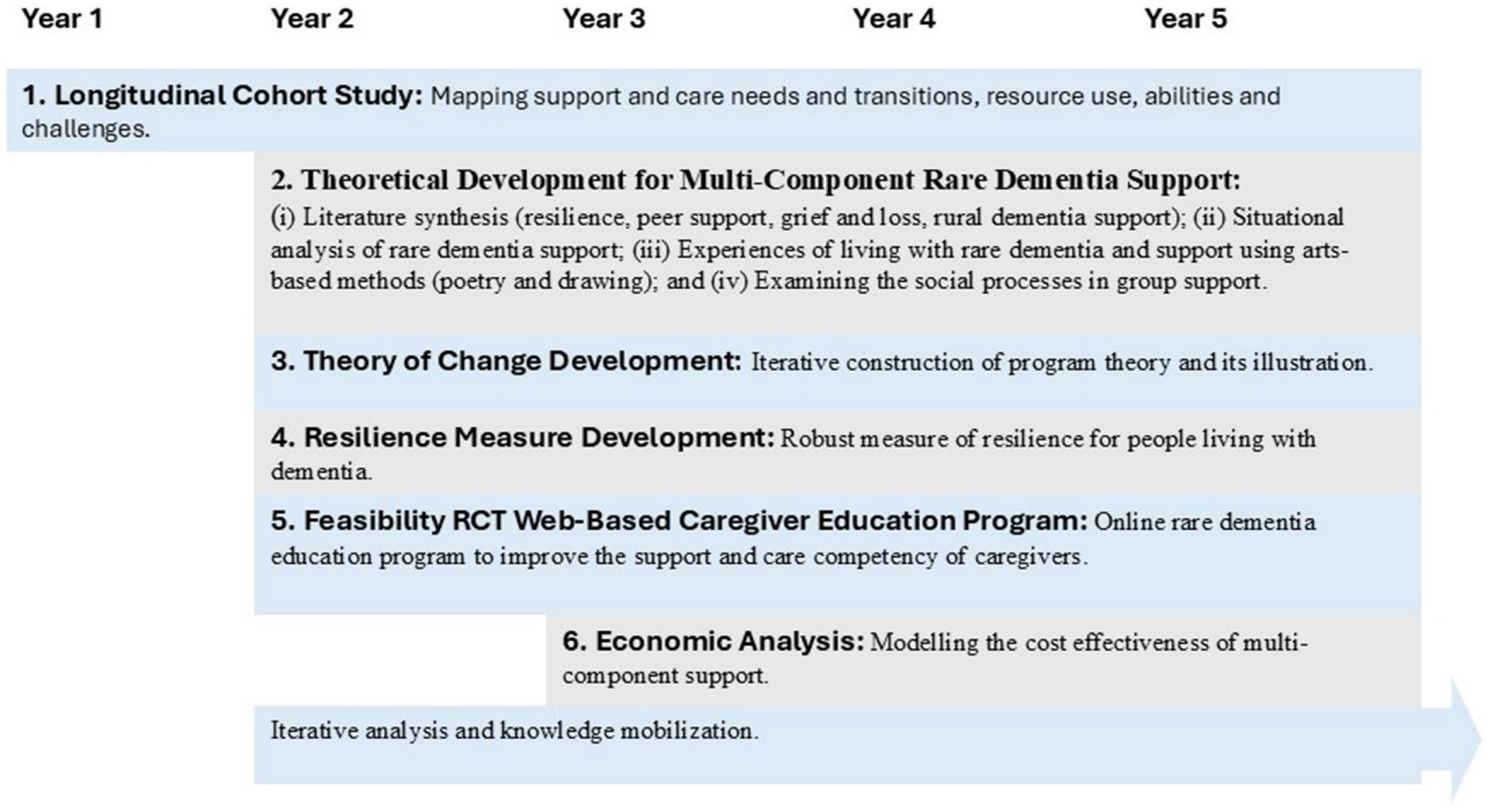
RDS impact study timeline and work streams.

As previously stated, the designing of an overview ToC for a multi-component support community was associated with a large longitudinal mixed method investigation. The RDS Impact Study aimed to add a substantive theoretical and methodological contribution to evidence demonstrating the role, impact and value of multi-component support for people affected by rare dementia. Presented in Figure 1, this study was comprised of multiple work streams and involved more than 800 unique participants living with a rare or young onset dementia or a family member in the UK and Canada.

The varied research areas and methods used were informing our understanding of the scope of need and pathways to and through various forms of support. For example, we completed:

- An integrative review of the literature on peer support for people living with dementia (Sullivan et al., 2022).
- A scoping review of dementia and people living in rural areas (Roberts et al., 2023) and a meta synthesis of grief and loss (Waddington et al., 2023).
- A situational analysis of rare dementia support to examine how rare dementia was situated within the complex organization of dementia care and support delivery (Sullivan et al., 2023).
- A cluster of studies utilizing innovative methods to provide an in-depth understanding of the experiences of living with a rare dementia (Camic et al., 2024; Rossi-Harries et al., 2024), language use in on-line peer support groups (Hayes et al., 2024) and the impact of group rare dementia support (Camic et al., 2025).
- A randomized controlled feasibility trial of a web-based caregiver educational program (Suarez-Gonzalez et al., 2023).
- Three phased studies to develop a conceptual model of resilience for people living with dementia (Roberts et al., 2025; Windle et al., 2023).

Our commitment to a participatory or co-produced approach meant that the both research participant and working group voices and consultation with our circle of relationships were paramount as we iteratively moved through the steps of theory development and design.

### 2.5 Theory of Change development process

The overall process for designing and refining the ToC and rare dementia support model is described in Table 1. Briefly, it involved a process whereby the working group first considered and achieved agreement on: (i) long-term outcomes; (ii) pathway to change; (iii) anticipated outcomes; (iv) strategies and resources to achieve the outcomes; and (v) assumptions that underpinned the context and strategies. And second, we undertook a method to refine and endorse the emerging model with internal and external representatives (Ghate, 2018).

**Table 1:** Theory of Change for rare dementia support development.

RDS working group process	Corresponding 6SQuID steps	Data sources
1. Defining the scope of rare dementia support need	1, 2	RDS Impact Study Work Streams 1–5 (Camic et al., 2024, 2025; Hayes et al., 2024; Roberts et al., 2023, 2025; Rossi-Harries et al., 2024; Suarez-Gonzalez et al., 2023; Sullivan et al., 2022, 2023; Waddington et al., 2023; Windle et al., 2023)
2. Mapping the design and desired impact (who and what)	3	Working Group Meetings
3. Mapping the mechanisms for change (how)	3, 4	Working Group Meetings
4. Describing underlying assumptions	3, 4	Working Group Meetings
5. Small scale testing	5	RDS Impact Study Work Streams 2, 4, 5 (Harding et al., 2023; Stevens-Neck et al., 2024; Waddington et al., 2022) RDS support delivery, observation and review
6. Consultation and refinement	5	Participatory consultation Working group meetings
7. Diagrammatic presentation of program theory	3, 4	Working group meetings
8. Evaluation and refinement^a^	6	RDS UK Direct Support Team RDS Canada Direct Support Team

a Further evaluation is ongoing.

The working group aimed to achieve broad consensus at each meeting as key elements and more complex issues were thoroughly discussed and at times challenged. Given related research activities were being implemented simultaneously, members of the working group were processing ideas and consulting with others in the external environment at all stages (i.e., validating alongside the emerging evidence). In addition, working group members who were practitioners were able to bring practice examples to meetings to illustrate a person or family navigating through the support design and the development of the mechanisms for change.

The mapping of the design, desired impact, mechanisms for change and underlying assumptions were informed by our investigations and more thoroughly examined during working group meetings. Central in our discussions was the development of a support model that worked effectively and efficiently within the current context of rarer forms of dementia being poorly understood, and complex and varied pathways to diagnosis and post-diagnostic support.

Small scale testing or further validation, conducted by members of the working group, was achieved in a variety of ways. By means of the support offered by RDS, we were able to generate small-scale observational explorations of on-line peer support groups (e.g., 7 groups with up to 12 participants) alongside two case reviews of individual support delivery.

During this stage we exchanged ideas and plans with respect to short and long-term evaluation. Focusing on group delivery at this stage, recommended evaluation questions and processes were set out in an evaluation protocol for support groups (Waddington et al., 2022). We also undertook an examination of social processes occurring within one group run by RDS that focused on independence and identity among people living with a rare dementia (Harding et al., 2023) and a mixed method evaluation of one support group addressing living with grief and loss (Stevens-Neck et al., 2024).

The illustration of the ToC underwent several iterations by the working group and consensus achieved before its use in participatory consultation meetings. We elaborate more on our external consultation strategy below.

### 2.6 Circle of relationships and responsibilities

We identified our responsibilities for participatory consultation to be varied and prioritized people with lived experience, current specialist RDS advisors, and external health and social care practitioners working in the dementia care sector. A draft narrative illustration and lay explanation of what a ToC is, and its purpose, was used to facilitate a series of direct consultation meetings. In Canada specifically, the meetings were facilitated by an Advisory Circle (N = 10) that had been established for the implementation of the RDS Impact Study and was transitioning into advising on the developing support model for the Canadian context. The Circle’s membership included practitioners, researchers and people affected by rare dementia. In the UK, meetings were conducted with an established research focus group which included people with lived experience and the RDS Advisory Committee (N = 12) made up of practitioners and dementia advocates. Finally, we were able to present the ToC to the RDS Direct Support Team (N = 6) and newly emerging RDS Canada team (N = 3). All consultation meetings were only loosely structured. They included advanced distribution of our lay document, a presentation of the ToC, followed by open discussion. Feedback was presented to the full working group, and no further changes were made to the ToC at that time.

## 3 Results

### 3.1 Navigating multiple data sources

As described above, our multiple data sources included evidence emerging from the research studies, experiential knowledge from support delivery, and conversational knowledge obtained through our consultation activities (Romão et al., 2023). Henceforth, we were able to align our understandings, often thematically, in relation to our values and assumptions, the “intervention” causal pathway, and expected outcomes. Table 2 sets out engagement in our consultation activities. We collected no demographic data on participants in these activities other than experiential and/or professional affiliations.

**Table 2:** Participatory consultation.

Year	UK	Total participants	Canada	Total participants
2021	Mixed consultation meetings^a^	1/10	Mixed consultation meetings	2/31^b^
2022	Mixed consultation meetings	4/30	Mixed consultation meetings	5/68

a Meetings were held with the RDS Advisory Committee and research Advisory Circle (Canada) which included people with lived experience, practitioners and practitioner scholars and the RDS Direct Support Teams.

b Number of meetings/total number of participants.

### 3.2 Theory of Change for a rare dementia support model

The vision for support, or long-term outcome, was agreed as follows: for anyone affected by, or at risk of a rare dementia, to have access to information, tailored support and guidance, and contact with others affected by similar conditions in a space of mutual respect and understanding. This outcome was underpinned by key values associated with building an accessible rare dementia support “community” as opposed to an intervention and were reflected in our assumptions in the ToC process. Consistent with our theoretical understandings and research findings, the focus was on the “relational” rather than “methods” as in a traditional intervention and permitted us flexibility to expand or add elements to the community at any time. We also shaped some of our ideas of relational work from relationship-centred dementia care or the Senses Framework (Ryan et al., 2008; Nwadiugwu, 2021; Watson, 2019), and Ruch (2018) and Ferguson et al. (2020) detailing of relationship-based social work. Accordingly, the rare dementia support community system and pathways to change were characterized by:

- Connection: Opportunities to meet and share with others affected by the same disease or condition in a respectful and safe place.
- Relational boundaries: A flexible relational triad (person living with dementia, their family, and the support team) acknowledging the spoken and unspoken, the visible and invisible, the conscious and the unconscious, and the contexts within which lives are lived.
- Shared learning and sharing solutions: Learning and education as a central element within all support exchanges.
- Agency: Recognizing that all members have agency, or exercise control over their decisions and actions (as on a continuum), and is supported in this context.
- “Being alongside”: Connection through both the “rare” and the ordinary and the practical.
- Continuous: Acknowledging that support and care needs change over time from symptom onset to bereavement and thus access to the support community is open.

Aiming to address many of the support gaps identified in the literature (Camic et al., 2024; Loi et al., 2023), the essential components or enablers of the community, or the “intervention”, included free in-person and virtual support and opportunities to participate in awareness raising initiatives, education and research activities. Connection was accessed by on-line membership registration followed by outreach by a support advisor. Members were neither admitted or discharged and accessed support mostly in a self-directed manner. The activities consistent with the ToC involved:

- Individual and family information and advice (e.g., co-construction of support goals with a support advisor).
- Small and large group support (i.e., tailored by disease, support relationship or topic).
- Educational seminars (e.g., sleep disorders, creative arts and well-being).
- Digital learning resources (e.g., online education program for people affected by primary progressive aphasia or posterior cortical atrophy).
- Practitioner education networks (e.g., speech-language therapists networking around language and communication symptoms and rehabilitation programs).
- Learning through research participation (e.g., studies exploring support and care, clinical trials).

**Figure 2:**
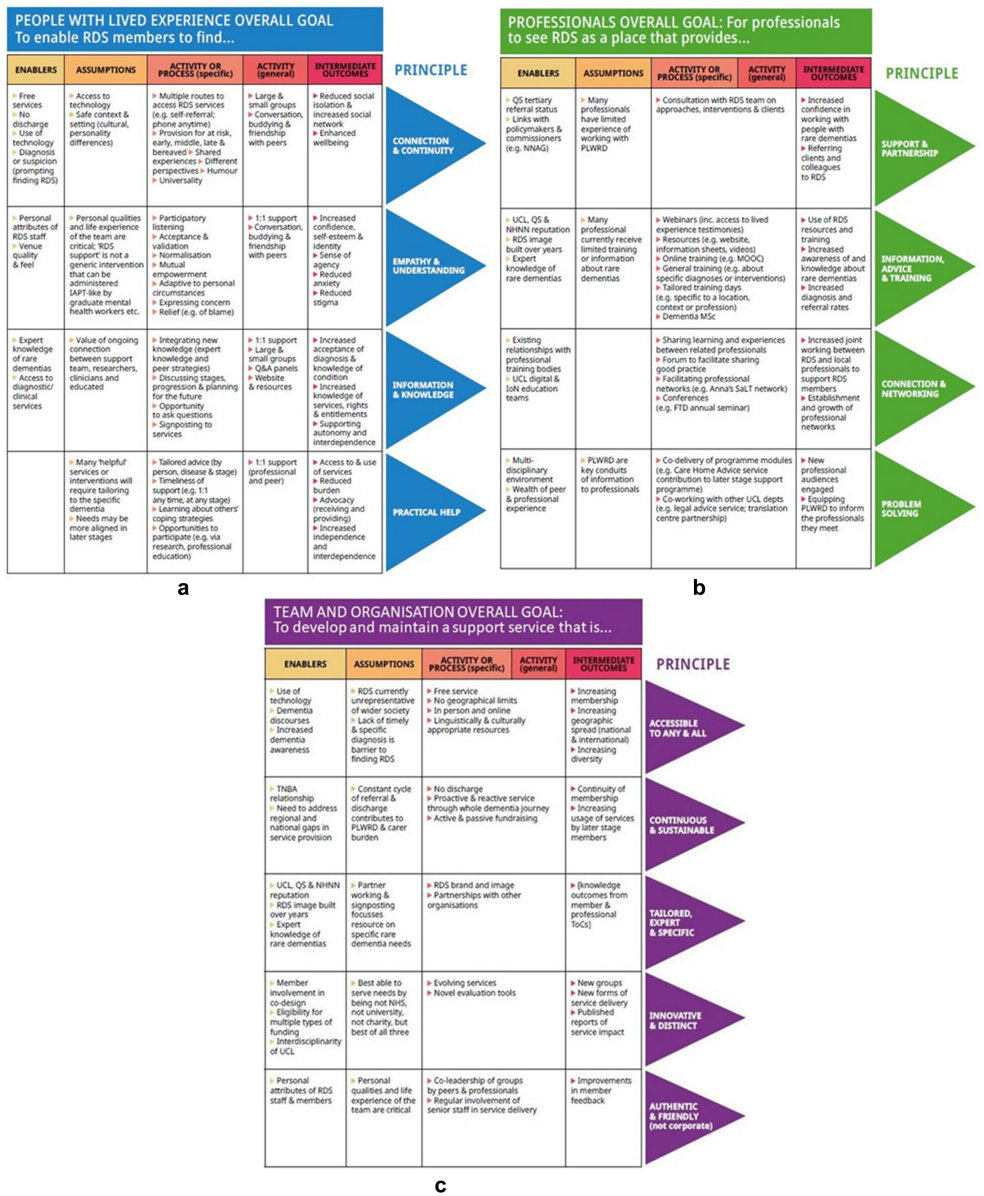
**(a)** Nested Theory of Change for people with lived experience. **(b)** Nested Theory of Change for professionals. **(c)** Nested Theory of Change for RDS team and organization.

Figures 2a–c illustrates a novel ToC consisting of 3 nested theories and each focused on our circle of relationships within the support community. The nesting of theories recognized that each of these may interact with each other in bringing about the desired results (Mayne, 2015). Noteworthy here was our use of principles rather than final goals or outcomes which were seemingly more fitting for an intervention with an end point. As stated previously, it was recommended that support be offered continuously, often over many years, where families transition through early, middle or later stages of decline and other life events and consequently support needs ebb and flow at different times. Whilst principles may appear incongruous with ToC, they ultimately acknowledged our community as a complex system and described our goals for change.

#### 3.2.1 People with lived experience

Figure 2a illustrates the overall principle for people diagnosed with a rare dementia or a family supporter which enabled community members to find connection and continuity, empathy and understanding, information and knowledge, and practical help. The activities or process towards the key goals reflected the concept of community through, for example, participatory listening, normalization and mutual empowerment or the integration of experiential and expert knowledge.

#### 3.2.2 Professional members

Professional members, also recognized as another key relationship for RDS members, were largely considered in relation to varied opportunities for education and discipline or interdisciplinary networking (Figure 2b). For practitioners or professionals, the principles were for these members to view the community as a place for support and partnership, information, advice and training, connection and networking, and new ideas or creative problem-solving. The key assumption with respect to professional members was the lack of rare dementia training and practice experience. Therefore, our focus on education and learning reinforced the value of tailored training, networked practice groups and shared support and care (joint working) among organizations and professionals.

#### 3.2.3 Support team or organization

The inclusion of the support team itself in our ToC is uncommon though consistent with our emphasis on community (Figure 2c). The principles here demonstrated an ongoing commitment to learning and development, but also the relational aspects of the support role – to develop a support service accessible to any or all, continuous (i.e., pre-diagnosis through to bereavement) and sustainable, tailored, expert and specific, innovative and distinct, and authentic and friendly. This additional nested theory was aimed at: (i) diminishing the expert – non-expert professional relationship and rather recognizing an ongoing relational triad; (ii) a commitment to continual learning through evaluation and research; and (iii) a responsibility to further partnership with local, national and international health and public health organizations to improve awareness and pathways to diagnosis and support and care.

### 3.3 Evaluation and sustainability

In a climate of cost containment and increasing health care costs, resources to deliver support were at the forefront of planning and consultation discussions. The cost–benefit analysis of our model was not fully completed owing to ongoing model revisions and the development of RDS Canada. As mentioned earlier, towards the latter stages of our ToC work funding to develop RDS Canada was received. Led by Nipissing University in Ontario, a 2-year grant for a proof-of-concept phase permitted a fortuitous opportunity to implement our model in a different setting and environment. Moreover, the partnership between RDS Canada and RDS also facilitated funding to conduct a realist evaluation to generate further knowledge pertaining to resources, localization with respect to geography and culture, and considerations for sustainability which is now underway.

## 4 Discussion

Within the ever-expanding scholarship addressing interventions to support individuals living with dementia and their families, there is much less attention on bringing evidence and theory to programming (Ghate, 2018) and how to both effectively and reasonably bring the voices of lived experience to such new initiatives (Walter et al., 2024). Whilst there is strong desire to understand “what works” in high quality dementia services, evaluation frameworks are often not sufficiently explicit to inform practitioners or program planners (Buetti and Bourgeois, 2024; Sullivan et al., 2022). And despite advancements in disease classifications and diagnosis, research on how to support the strengths and needs of people living with diverse dementia syndromes is also underdeveloped.

This paper presented the development of a ToC for a support model for people living with or affected by rare dementia. Responding to policy demands for improved systems of support and care for people living with dementia (Weidner et al., 2024) and informed by a comprehensive study on supporting people with rare dementia (Brotherhood et al., 2020), we proposed a nested ToC for a model of rare dementia support. Notably, the ToC addressed the intersection among people with lived experience, practitioners working with this service user group in other sectors, and an innovative consideration of the rare dementia specialist support team for program impact. Co-designed by an interdisciplinary team of practitioner scholars with people with lived experience, the ToC illustrated pathways to and through activities for continuous and tailored support and the overarching principles, or longer-term goals, that underpin these.

Recognizing the complexities within the lives of people living with dementia, their multiple and the non-linear care and support transitions over time, and varied pathways within constrained health and social care sectors, the model was characterized as a community. Rufuting it as an intervention, the community’s emphasis was on its relational features that facilitated agency, normalization, shared support, learning and problem-solving. Although a unique initiative, Aguzzoli Peres et al. (2024) recently drew on similar values in building a community through their Walking the Talk for Dementia collaboration and “building an ecosystem: connecting for thriving” (p. 2314). Further recognizing individual and system complexities, the community was also characterized by a series of principles rather than distinctive outcomes. The multi-component model of varied supports, closely aligned to education and learning through research, also positively enabled advocacy efforts intended for responsive systems for care and support and knowledge mobilization through the community itself.

At present, the support community is situated within a university research centre (although closely integrated with the National Health Service) and delivered by a team of support advisors with interdisciplinary contributions from practitioner scholars (e.g., psychologists, nurses, speech-language therapists and neurologists). Outreach by higher education institutions is not new but models of engagement have varied. Having already extended rare dementia leadership beyond the boundaries of the university to build connections with and on behalf of people affected for some time, RDS presented itself as a critical opportunity (Weerts and Sandmann, 2024). Collective empowerment amongst “experiential experts”, community-based organizations, scientists and other scholars, and a commitment to knowledge exchange and mobilization vis-á-vis this community-university innovation, informed a support model that would be maintained by an ongoing looped portfolio of knowledge production, support solutions and education opportunities (Hidayat and Stoecker, 2021). A position of being “not quite health care, not quite a dementia charity and not quite a university” also capitalized on community-engaged scholars as mavericks in the ever-changing environments of higher education, and health and social care (Sullivan et al., 2023). The concept of boundary-spanning within higher education, bridging the gap between academia and community and cultivating partnered co-production to activate change or solutions, is relevant here (Dienno et al., 2024; Weerts and Sandmann, 2024). Whilst location within the higher education sector draws benefits from its education and research activities, we recognize this may not be feasible or ideal in other locations. Given our recommending the close alignment to education and research for capacity strengthening and knowledge mobilization, it is possible that varied organizational partnerships, valuing innovation and scholarship, could construct a similar model.

The model is easily accessible by virtue of it being free at point of entry. Furthermore, for those living in rural or remote regions all support is available virtually. This is tremendously important given the strong desire among people to connect with others with a similar diagnosis (Camic et al., 2024; Harding et al., 2023). In terms of diversity, equity and inclusion, we are currently undertaking various activities to ensure barriers to accessing support are addressed. This includes staff training (e.g., culturally safe support), translation of support materials to different languages, partnerships with organizations already engaged with multi-cultural or newcomer communities, and co-learning with respect to the experiences of living with dementia among diverse groups and the intersection of multiple identities.

Finally, the ToC provides a communication tool given RDS’s unique leadership voice at community-wide and other strategic planning for people affected by dementia and, importantly, with potential funders. We value this tool in our context of universal health care and shifting political and social environments. Dementia support and care increasingly relies on the philanthropic sector which, in turn, is progressively becoming evidence-based philanthropy (Greenhalgh and Montgomery, 2020). Whilst government funds are accompanied with tighter controls, a reliance on charitable funds brings about other challenges. Thus, our ability to communicate impact and utilize the ToC to continue to build and mobilize new knowledge is a fundamental responsibility within our work.

### 4.1 Strengths and limitations

Our detailing of a conceptual framework or ToC for rare dementia support positively responds to a call for innovative models of support and quality improvement in programming for people living with dementia (Farina et al., 2017; Lord et al., 2022). The support model’s specific strength lie in its focus on people affected by young onset or atypical dementia given they are often overlooked in strategic planning for systems of care for people living with dementia. Beneficially, the theoretically and evidence-informed ToC provides a blueprint for intervention evaluations and ongoing quality monitoring, expedited RDS development in other jurisdictions or to inform the development of similar ToC to inform other rare dementia services and beyond (e.g., other neurological diseases). The identification of the criticality of community and relationship may be an important consideration for future intervention development because it moves practice beyond the narrower focus on method or technique. The community, its characteristic principles and varied activities are also sufficiently flexible to enable modifications as new information comes to bear for the support service either through our ongoing research or as broader systems shift in response to governmental initiatives (Ghate, 2018).

Work undertaken to develop a ToC is both time consuming and multifaceted. Whilst our approach and the processes followed align with existing literature, it is difficult to articulate how the researchers thought about our new discoveries and other learning and mapping this on various iterations of the ToC. We do, however, acknowledge that this work is dynamic and will continue to evolve.

We also recognize that simplifying mechanisms for change for what is actually quite complex could lead to misunderstandings given our emphasis on a non-linear support model and potentially minimize the important role this model has for people affected. This too could lead to unrecognized modifications or “program drift” (Ghate, 2018).

## 5 Conclusion

The RDS community offers a new contribution to some of the pressing challenges within the domain of dementia care and support for people affected by dementia. Its characteristic features, largely centred on connection and relationship, offer an alternative to conventional dementia support where RDS is tailored by condition, age, stage and family status. Its unique location within the university sector maximizes opportunities to strengthen its looped portfolio of support, education and research activities.

## Data Availability

The raw data supporting the conclusions of this article will be made available by the authors, without undue reservation.
